# Resting-state MRI functional connectivity as a neural correlate of multidomain lifestyle adherence in older adults at risk for Alzheimer’s disease

**DOI:** 10.1038/s41598-023-32714-1

**Published:** 2023-05-09

**Authors:** Meishan Ai, Timothy P. Morris, Jiahe Zhang, Adrián Noriega de la Colina, Jennifer Tremblay-Mercier, Sylvia Villeneuve, Susan Whitfield-Gabrieli, Arthur F. Kramer, Maiya R. Geddes, for the PREVENT-AD Research Group, Paul Aisen, Elena Anthal, Melissa Appleby, Pierre Bellec, Fatiha Benbouhoud, Véronique Bohbot, Jason Brandt, John C. S. Breitner, Céline Brunelle, Mallar Chakravarty, Laksanun Cheewakriengkrai, Louis Collins, Doris Couture, Suzanne Craft, Mahsa Dadar, Leslie-Ann Daoust, Samir Das, Marina Dauar-Tedeschi, Doris Dea, Nicole Desrochers, Sylvie Dubuc, Guerda Duclair, Marianne Dufour, Mark Eisenberg, Rana El-Khoury, Pierre Etienne, Alan Evans, Anne-Marie Faubert, Fabiola Ferdinand, Vladimir Fonov, David Fontaine, Renaud Francoeur, Joanne Frenette, Guylaine Gagné, Serge Gauthier, Valérie Gervais, Renuka Giles, Julie Gonneaud, Renee Gordon, Claudia Greco, Rick Hoge, Louise Hudon, Yasser Ituria-Medina, Justin Kat, Christina Kazazian, Stephanie Kligman, Penelope Kostopoulos, Anne Labonté, Marie-Elyse Lafaille-Magnan, Tanya Lee, Jeannie-Marie Leoutsakos, Illana Leppert, Cécile Madjar, Laura Mahar, Jean-Robert Maltais, Axel Mathieu, Sulantha Mathotaarachchi, Ginette Mayrand, Melissa McSweeney, Pierre-François Meyer, Diane Michaud, Justin Miron, John C. Morris, Gerhard Multhaup, Lisa-Marie Münter, Vasavan Nair, Jamie Near, Holly Newbold-Fox, Nathalie Nilsson, Véronique Pagé, Tharick A. Pascoal, Mirela Petkova, Cynthia Picard, Alexa Pichet Binette, Galina Pogossova, Judes Poirier, Natasha Rajah, Jordana Remz, Pierre Rioux, Pedro Rosa-Neto, Mark A. Sager, Eunice Farah Saint-Fort, Mélissa Savard, Jean-Paul Soucy, Reisa A. Sperling, Nathan Spreng, Frederic St-Onge, Christine Tardif, Louise Théroux, Ronald G. Thomas, Paule-Joanne Toussaint, Miranda Tuwaig, Etienne Vachon-Presseau, Isabelle Vallée, Vinod Venugopalan, Karen Wan, Seqian Wang

**Affiliations:** 1 Department of Psychology, Northeastern University, Boston, MA USA; 2 Department of Physical Therapy, Movement and Rehabilitation Sciences, Northeastern University, Boston, MA USA; 3 Department of Neurology and Neurosurgery, Faculty of Medicine, McGill University, Montreal, QC Canada; 4 STOP-AD Centre, Centre for Studies on Prevention of Alzheimer’s Disease, Montreal, QC Canada; 5 Douglas Mental Health University Institute Research Centre, Affiliated with, McGill University, Montreal, QC Canada; 6 Department of Psychiatry, McGill University, Montreal, QC Canada; 7 Beckman Institute for Advanced Science and Technology, University of Illinois at Urbana Champaign, Urbana-Champaign, IL USA; 8 Montreal Neurological Institute, Montreal, QC Canada; 9 Alzheimer’s Therapeutic Research Institute at University of Southern California, San Diego, CA USA; 10 Université de Montréal, Montreal, QC Canada; 11 John Hopkins University, Baltimore, MD USA; 12 Research Centre for Studies in Aging, McGill University, Montreal, QC Canada; 13 Department of Biomedical Engineering, McGill University, Montreal, QC Canada; 14 Wake Forest School of Medicine, Winston-Salem, NC USA; 15 McConnell Brain Imaging Center, McGill University, Montreal, QC Canada; 16 School of Population and Global Health, McGill University, Montreal, QC Canada; 17 Department of Psychology, McGill University, Montreal, QC Canada; 18 Neuroscience Department, McGill University, Montreal, QC Canada; 19 Washington University School of Medecine in St-Louis, St. Louis, MO USA; 20 Department of Pharmacology, McGill University, Montreal, QC Canada; 21 Wisconsin Alzheimer’s Institute, UW School of Medicine and Public Health, Milwaukee, WI USA; 22 Center for Alzheimer’s Research and Treatment Harvard Medical School, Boston, MA USA; 23 School of Medicine, University California, San Diego, La Jolla, CA USA; 24 Northwestern University, Chicago, IL USA

**Keywords:** Cognitive ageing, Lifestyle modification

## Abstract

Prior research has demonstrated the importance of a healthy lifestyle to protect brain health and diminish dementia risk in later life. While a multidomain lifestyle provides an ecological perspective to voluntary engagement, its association with brain health is still under-investigated. Therefore, understanding the neural mechanisms underlying multidomain lifestyle engagement, particularly in older adults at risk for Alzheimer’s disease (AD), gives valuable insights into providing lifestyle advice and intervention for those in need. The current study included 139 healthy older adults with familial risk for AD from the Prevent-AD longitudinal aging cohort. Self-reported exercise engagement, cognitive activity engagement, healthy diet adherence, and social activity engagement were included to examine potential phenotypes of an individual’s lifestyle adherence. Two adherence profiles were discovered using data-driven clustering methodology [i.e., Adherence to healthy lifestyle (AL) group and Non-adherence to healthy lifestyle group]. Resting-state functional connectivity matrices and grey matter brain features obtained from magnetic resonance imaging were used to classify the two groups using a support vector machine (SVM). The SVM classifier was 75% accurate in separating groups. The features that show consistently high importance to the classification model were functional connectivity mainly between nodes located in different prior-defined functional networks. Most nodes were located in the default mode network, dorsal attention network, and visual network. Our results provide preliminary evidence of neurobiological characteristics underlying multidomain healthy lifestyle choices.

## Introduction

The number of individuals on the Alzheimer’s disease (AD) continuum, from preclinical AD to AD dementia, is currently estimated at about 416 million globally^1^. AD presents a significant impact on public health systems and the well-being of older adults and caregivers^2^. Therefore, it is crucial to identify prevention strategies to prevent or delay disease progression. Adherence to healthy lifestyle habits may reduce the risk of dementia and preserve cognitive health in at-risk seniors, in a cost-effective way. Multidomain lifestyle behaviors may prevent or delay up to 40% of dementias^3^. Previous studies have found that healthier diet habits^4^, physical activity engagement^5^, cognitively stimulating activity participation^6^, and social support^7^ prevented or delayed cognitive decline among healthy older adults. Similar lifestyle habits (e.g., engaging in exercise, healthy diet, and cognitive activity) are also associated with decreased risks of developing mild cognitive impairment (MCI) and AD^8–10^. Therefore, promoting healthy lifestyle behaviors are important for brain resilience and AD prevention.

Most prior studies focused on a single lifestyle variable. A few studies examined multidomain lifestyles, cognitive and brain health but with a limited number (i.e., < 3) of lifestyle variables^11–13^. However, lifestyle behaviors may not act on the brain health in isolation. According to previous reviews, intervention for AD and cognitive impairment prevention is moving from targeting a single lifestyle factor to multidomain lifestyle features in recent decades, as multidomain interventions mimic a more ecologically valid approach to voluntary adherence to modifiable lifestyles habits^14,15^. This approach is also based on the assumption that AD is a complex disorder that is associated with multiple risk and protective factors^16^. Thus, older adults at risk for AD may benefit more from interventions that target multiple factors at once. These reasons underscore the need to deepen our understanding of engagement in multidomain lifestyle factors. How individuals adhere to multidomain lifestyles voluntarily in a real-world setting is presently unknown. Only a few behavioral studies have examined this question. For example, more social support was associated with more physical activity engagement and healthy food intake^17^. Additionally, there was a reverse relationship between physical activity engagement and dietary fat^18^. Therefore, we hypothesize that engagement in each lifestyle factor is not entirely independent (i.e., people who adhere to one healthy habit might be more or less likely to adhere to another). By identifying potential lifestyle profiles among individuals, we will be able to provide insights into personalized lifestyle promotion interventions.

The brain systems underlying successful multidomain healthy lifestyle engagement are presently poorly understood. This knowledge is critical to more effectively design personalized lifestyle interventions. A few studies to date have investigated the relationship between multidomain lifestyle behaviors and brain health. Bittner et al. (2019) found that a higher combined lifestyle risk score (i.e., defined by physical activity, social engagement, alcohol intake, and smoking) was associated with altered functional connectivity and gyrification in motor and frontal areas in healthy older adults. Relatedly, individuals with higher lifestyle risk scores showed an older brain age, estimated by T1-weighed structural images^19^. Multiple lifestyle factors were also associated with neuropathological biomarkers (e.g., amyloid burden)^13,20^. Prior research has focused on brain characteristics as the outcome of healthful lifestyles, rather than the cause. This overlooks the bidirectional relationship between the brain and lifestyle habits. Morris et al. (2022) found that functional connectivity in regions related to inhibitory control predicted older adults’ sedentary behaviors change after an intervention. Therefore, it may be equally important to consider the potential neural features that impact an individual’s lifestyle choices when interpreting the findings in cohort studies where directionally can be hard to discern. Additionally, the majority of these studies focused on generally healthy older populations, where these findings may not generalize to those at risk for AD and who stand to benefit the most from lifestyle engagement. Relatedly, there is a need for a mechanistic understanding of the cognitive processes and neural substrates that support intervention response in individuals at risk for AD to provide a deeper understanding underlying the efficacy of interventions^21^.

In the current study, we aim to (1) identify the potential profiles of individuals’ lifestyle habits based on healthy diet adherence, exercise engagement, cognitive activity engagement, and social network, and (2) identify the dissociable neurobiological substrates among lifestyle phenotypes based on functional and structural brain imaging. We examined these research questions in a well-characterized high AD-risk population from the Pre-symptomatic Evaluation of Experimental or Novel Treatments for Alzheimer’s disease (PREVENT-AD) longitudinal cohort^22^. Given the heterogeneity of results and distinct methodologies employed in previous studies, we chose to use a data-driven approach to identify lifestyle phenotypes and the underlying neural distinctions.

## Method

### Participants

We obtained cross-sectional data from 139 cognitively healthy older adults, as a subsample from the Presymptomatic Evaluation of Experimental or Novel Treatments for Alzheimer’s disease (PREVENT-AD) cohort. This longitudinal cohort at McGill University, recruited participants who were cognitively normal but are at an increased risk of developing dementia and have an immediate family member with a history of AD. Selection criteria for the 139 sub-sample included cognitive status, data completeness and quality. Participants with potential cognitive impairment, evaluated by a neuropsychologist, were not included in this sub-sample. Demographic information (age, sex, years of education, and APOE genotype) was collected during the baseline visit of each participant, which occurred between 2011 and 2017. APOE genotype was labeled as whether individuals were heterozygous carriers for alleles ε3 and ε4 or not. The consent form was reviewed and signed by all participants from this study. Specific consent forms were obtained from participants prior to each experimental procedure. The consent form, protocols and study procedures were approved by the McGill Institutional Review Board and/or Douglas Mental Health University Institute Research Ethics Board. All procedures were carried out in compliance with the ethical principles of the Declaration of Helsinki.

### Behavioral data

Psychosocial and lifestyle questionnaires were administered through Qualtrics from 2017 to 2019 at separate timepoints (https://www.qualtrics.com). Because the majority of lifestyle variables were collected in 2018, the age of each individual was all corrected to their age in 2018. Below is the specific information about the lifestyle, psychosocial, and cognitive measures that were used in the current study.

#### The lifetime total physical activity questionnaire

Exercise and Sports sub-scale. This questionnaire was developed and validated by Friedenreich et al. 1998. The participants reported the frequency of each type of physical activity they engaged in by day, week, month, and year, and the duration of each activity per session. The accumulated time spent in each activity per year was calculated and summed across all activities, in order to derive the total time in exercise and sports engagement in a year. The total score was used as an indicator of physical activity engagement of individuals. The scale was assessed throughout the year 2017.

#### The lifetime cognitive activity scale

This scale was developed and validated by Wilson et al. 2003. Participants reported the frequency of different cognitive activities in different age stages: 6, 12, 18, 30–40, 40 years old and currently. The present study focuses on current cognitive engagement only. The sub-scale of current cognitive activity engagement lists 11 activities, and the participants indicated the frequency for each activity on a Likert scale from 1 (once per year) to 5 (every day). The sum score for this sub-scale was calculated as an indicator of cognitive activity engagement for individuals. The scale was assessed in 2018.

#### The diet habit questionnaire

This questionnaire consists of 4 items from the Mediterranean Diet Adherence scale (Martínez-González et al. 2012) and 22 items from the Educoeur questionnaire diet sub-scale (Goyer et al. 2013). Red meat intake was removed from the analysis because of a large portion of missing values. Higher scores suggest higher adherence to recommended dietary habits across all items. The summed score was calculated as an indicator of adherence to a healthy diet. The scale was assessed throughout the year 2018.

#### The social life frequency scale

This in-house questionnaire contains four items asking about the frequency of getting together with friends/relatives, inviting friends/relatives to participants' homes, visiting friends/relatives at their homes, and on the telephone with friends/relatives over the past month. The participants rate each item on a Likert scale from 1 (Not at all in the past month) to 6 (Everyday). The sum score across 4 items was calculated as an indicator of social activity engagement. The scale was assessed in 2018.

#### The geriatric depression scale (GDS)

This questionnaire was developed and validated^23^ to assess depression symptoms in older adults. The 15-item version was used. For each item, participants responded with yes or no. The maximal score is 15 and a higher score indicates a greater level of depression.

#### The geriatric anxiety inventory (GAI)

This questionnaire was developed and validated^24^ to assess anxiety symptoms in older adults. The 20-item version was used. For each item, participants responded with agree or disagree. The maximal score is 20 and a higher score indicates a greater level of anxiety.

#### Stress subscale from the depression anxiety stress (DASS)

This subscale was developed and validated^25^ for assessing stress. This subscale has 14 items, each on a Likert scale from 0 to 3. A higher score indicates a greater level of stress.

#### Subscale from the psychological wellbeing scale

This scale was developed and validated^25^ and the subscale to assess purpose of life was administered in the current study. This subscale has 14 items, each on a Likert scale from 1 (Strongly agree) to 6 (Strongly disagree). Higher score indicates greater level of purpose of life.

#### The apathy evaluation scale (AES)

This questionnaire was developed and validated^26^ to assess amotivation across cognitive, behavioral and emotional domains. The questionnaire contains 20-items. For each item, participants responded on a scale from 1 (A lot) to 4 (Not at all). The maximal score is 80 and higher score indicates more apathy.

#### The big five inventory

This inventory was developed and validated^27^ to assess five personality dimensions: Extraversion, Neuroticism, Consciousness, Agreeableness, and Openness. The questionnaire has 44 items in total, participants responded to each by rating from 1 (disagree strongly) to 5 (agree strongly). The summary scores for all five dimensions were calculated separately.

#### The repeatable battery for the assessment of neuropsychological status (RBANS)

This neuropsychological battery was developed with five cognitive domains: Immediate Memory (i.e., list learning, story remembering), Visuospatial Ability (i.e., figure copy, line orientation), Language (i.e., picture naming, semantic fluency), Attention (i.e., digit span, coding), and Delayed Memory (i.e., list recognition, story recall, figure recall)^28^. The battery was administered annually to all participants enrolled in PREVENT-AD. Data from 2016 and 2019 were included in the current analysis and change scores were computed by subtracting performance in 2016 from 2019, in order to assess the longitudinal change in cognitive function over time. There are 22 out of 139 participants had incomplete RBANS data, therefore 117 participants were included in the analysis.

### MRI data acquisition and preprocessing

Participants were scanned in a Siemens TIM Trio 3 Tesla magnetic resonance imaging (MRI) scanner. The Siemens standard 12-channel coil was used. T1-weight structural data were acquired using an MPRAGE (Magnetization Prepared Rapid Gradient Echo Imaging) sequence. The parameters were as follows: repetition time (TR) = 2300 ms, echo time (TE) = 2.98 ms, inversion time (TI) = 900 ms, flip angle = 9°, Field of View (FOV) = 256 × 240 × 176 mm, phase encode A-P, GRAPPA acceleration factor = 2. Resting-state functional MRI data were acquired by an echo-planar imaging (EPI) sequence. The scanning duration for each run was 5.04 min and two runs were performed sequentially. The parameters were as follows: repetition time (TR) = 2000 ms, echo time (TE) = 30 ms, flip angle = 9°, FOV = 256 × 256 × 252 mm, phase encode A-P, Bandwidth = 2442/px. Thirty-two slices were collected in each run.

The preprocessing of both functional and structural data was performed using the fMRIprep pipeline^29^. The structural images went through skull stripping, brain tissue segmentation, spatial normalization to Montreal Neurological Institute (MNI) space, and surface reconstruction. Preprocessing steps for the resting-state functional images include head motion correction, realignment, slice timing correction for sequential acquisition, susceptibility distortion correction, co-registration to reconstructed structural images, and spatial normalization to standard space. Some extra preprocessing steps were performed on functional images in CONN toolbox^30^ as follows. The functional data were smoothed using a full-width half-maximum kernel of 6 mm. Volumes with framewise displacement above 0.5 mm and/or global blood-oxygen-level-dependent (BOLD) signal changes above 3 standard deviations were flagged as motion outliers. Noise reduction was performed with 10 noise components from white matter and cerebrospinal areas estimated using an anatomical component-based noise correction procedure (aCompCor) (Behzadi et al. 2007), 12 estimated subject-motion parameters estimated from fMRIprep, motion outliers, and constant and first-order linear session effects detected in CONN. Nine participants were removed because of having less than five minutes of scanning time after the outlier scans being removed^31^. These nine participants were not included in any analysis of the current study.

### Structural and functional image features preparation

Schaefer 100 parcellation was applied to both functional and structural brain data^32^. Cortical area and thickness were extracted from the 100 parcels in T1 structural images, which resulted in 200 structural features. Fisher-Z transformed bivariate Pearson correlation coefficients between each pair of parcels were calculated in CONN toolbox for the resting state functional images, which resulted in (100 × 100 − 100)/2 = 4950 functional features.

### Lifestyle phenotyping (K-means clustering)

The k-means algorithm was used to cluster the participants into different lifestyle phenotypes. K-means is an unsupervised methodology to investigate patterns in a dataset, by defining a priori the potential number of clusters (*k*) that exist in the dataset. For a given k, the algorithm will assign each data point into a cluster by minimizing the distance between that data point and the centroid (i.e., the center of a cluster). All data were transformed to z-scores initially and all analysis was done in R (version 4.1.3). The four lifestyle variables (physical activity, cognitive activity, diet, and social activity) were separately entered into a linear regression model as dependent variables, with age, years of education, sex, and APOE4 carrier status as independent variables. Residuals from the regression models of the four lifestyle variables were entered into *k-means* function from the *stats* package, with number of clusters set from 1 to 9. The *Clusterboot* function from *fpc* package was used to calculate the Jaccard index by bootstrapping the clustering 100 times and calculating how many datapoints stayed in the same cluster across resamples. The Jaccard index was calculated by the ratio between number of data points assigned to the same cluster and the total number of data points. We determined the optimal *k* using two criteria: 1) highest Jaccard index, a higher value indicates higher stability, and 2) ‘elbow’ for within sum of squares for *k* versus *k* + 1, a lower value of within sum of squares indicates higher compactness for a cluster, which is the distance of each participant from the centroid.

The final chosen clusters were validated by examining group differences in psychosocial variables and longitudinal cognitive changes. The goal of this step is to confirm that the resultant clusters are derived from real differences in the data rather than being spurious. Psychosocial variables included depression, anxiety, stress, apathy, purpose of life, and Big Five Inventory. Cognitive measures included the change in scores from 2019 to 2016 across the five dimensions of the RBANS battery. False Discovery Rate (FDR; *p* < 0.05) correction was applied within dimensions of Big Five Inventory, RBANS, and other psychosocial variables (i.e., depression, anxiety, stress, apathy, purpose of life) separately. A t-test was performed to examine the difference between cluster groups.

### Imaging classification

We ran a classification analysis using neuroimaging features to classify groups obtained in the k-means clustering solution with optimal *k.* Age, sex, educational years, APOE4 genotype, and mean head motion (only for the functional features) were regressed out of each feature and the residuals were used in the prediction model, in order to control for potential confounding. To select the imaging feature set that would be used in the training model, a feature selection filter was applied to all imaging features using the *sbf* function in the *caret* package. For each imaging feature, a logistic regression model was generated with the clustering solution as a binary categorical dependent variable, and the imaging feature as an independent variable. Then the features that predicted the clustering at *p* < 0.05 in the logistic models were selected across a tenfold cross-validation manner.

The main classification model was built using a linear support vector machine (SVM) classifier with linear kernel in *kernlab* package. The linear SVM classifier tries to find a hyperplane that separates the data points in a N-dimension space (*N* = the number of features in this model). The analysis was done in a nested cross-validation manner, with 25 inner loop resampling and tenfold outer loop cross-validation, in order to avoid over-fitting when using the same samples for tuning and testing. The tenfold in the outer loop was consistent with the folds in feature selection described above. A list of cost C was created varying from 0.25 to 64. The analysis involved two steps: (1) Tuning the parameter cost in inner loop resamples by maximizing Area Under Curve (AUC) in a receiver operating characteristics (ROC) curve, (2) testing the model by applying the best cost value for each fold in outer loop cross-validation. Model performance was averaged across all 10 folds in the outer loop. The same pipeline with the same fold splits was repeated in the same seed with a Random Forest model and a SVM classifier with non-linear kernel, in order to examine the stability of the outcome across models. Number of predictors to be sampled at each split, and the minimum number of data points in a node for further splitting were tuned in the inner resample. The number of trees was set at 1000.

The performance of each classification model was evaluated by accuracy, sensitivity, specificity, and AUC. An AUC of 1 indicates perfect prediction, and an AUC of 0.5 performs at chance. The significance of accuracy was examined via a permutation test. The sample was permutated and generate a new accuracy which repeated 1000 times, in order to produce the null distribution of the accuracy. The *p* value was determined by the percentage of values from the null distribution that are equal or greater than the observed accuracy.

**Figure 1:**
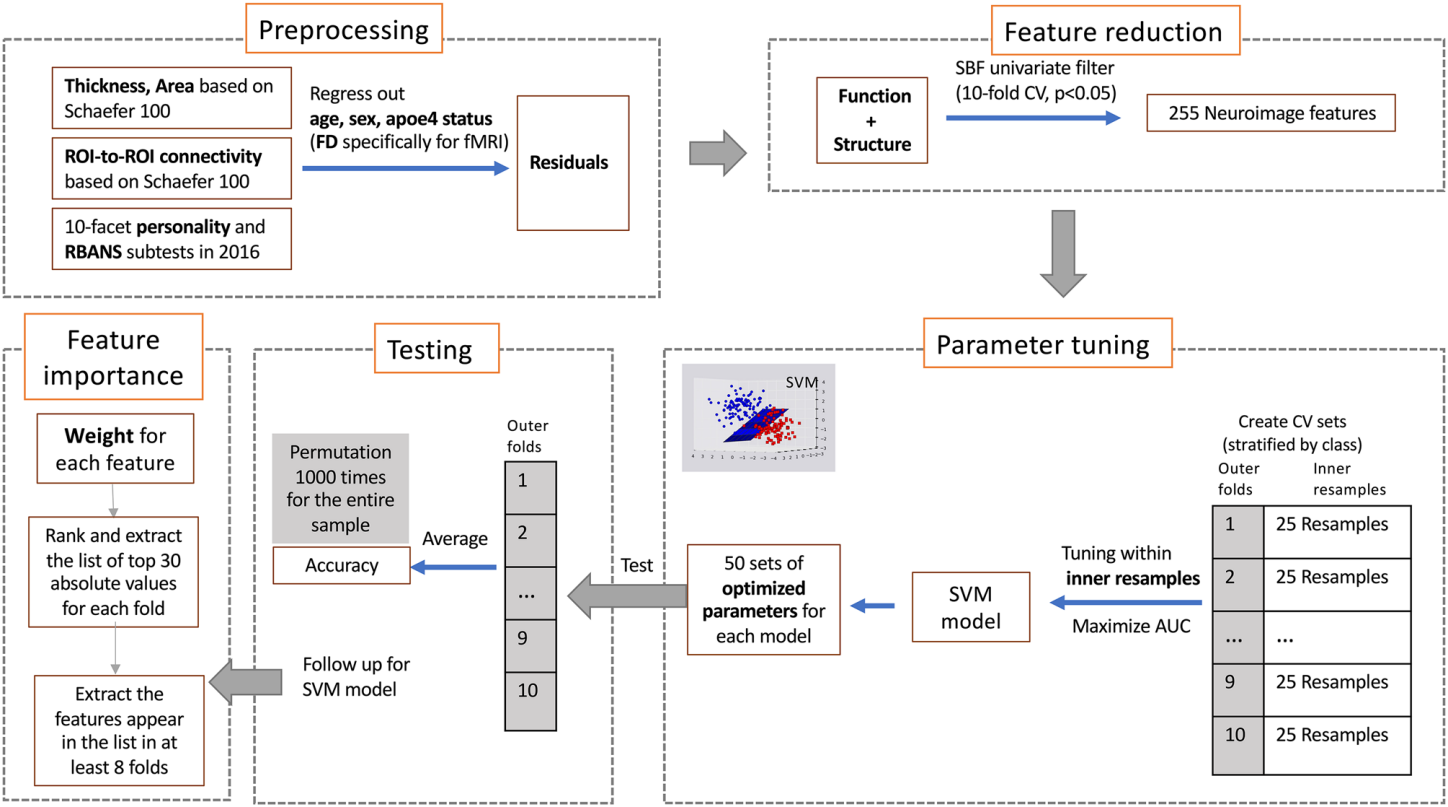
The classification analysis pipeline.

The whole schema for the analysis pipeline is depicted in Fig. 1:

### Pos-hoc analysis

In order to determine which neurobiological features were important for the classification of individuals into lifestyle adherence clusters, the weights for all features were extracted from the linear SVM model. Specifically, coefficients from the orthogonal vector to the hyperplane were extracted and the greater absolute value of the coefficient for each feature indicates greater importance in separating the clusters. We ranked the absolute values of coefficients and selected the features that ranked among the top 30 for no fewer than 8 out of the 10 outer folds as the final list of important features.

## Results

### Descriptive information

**Figure 2:**
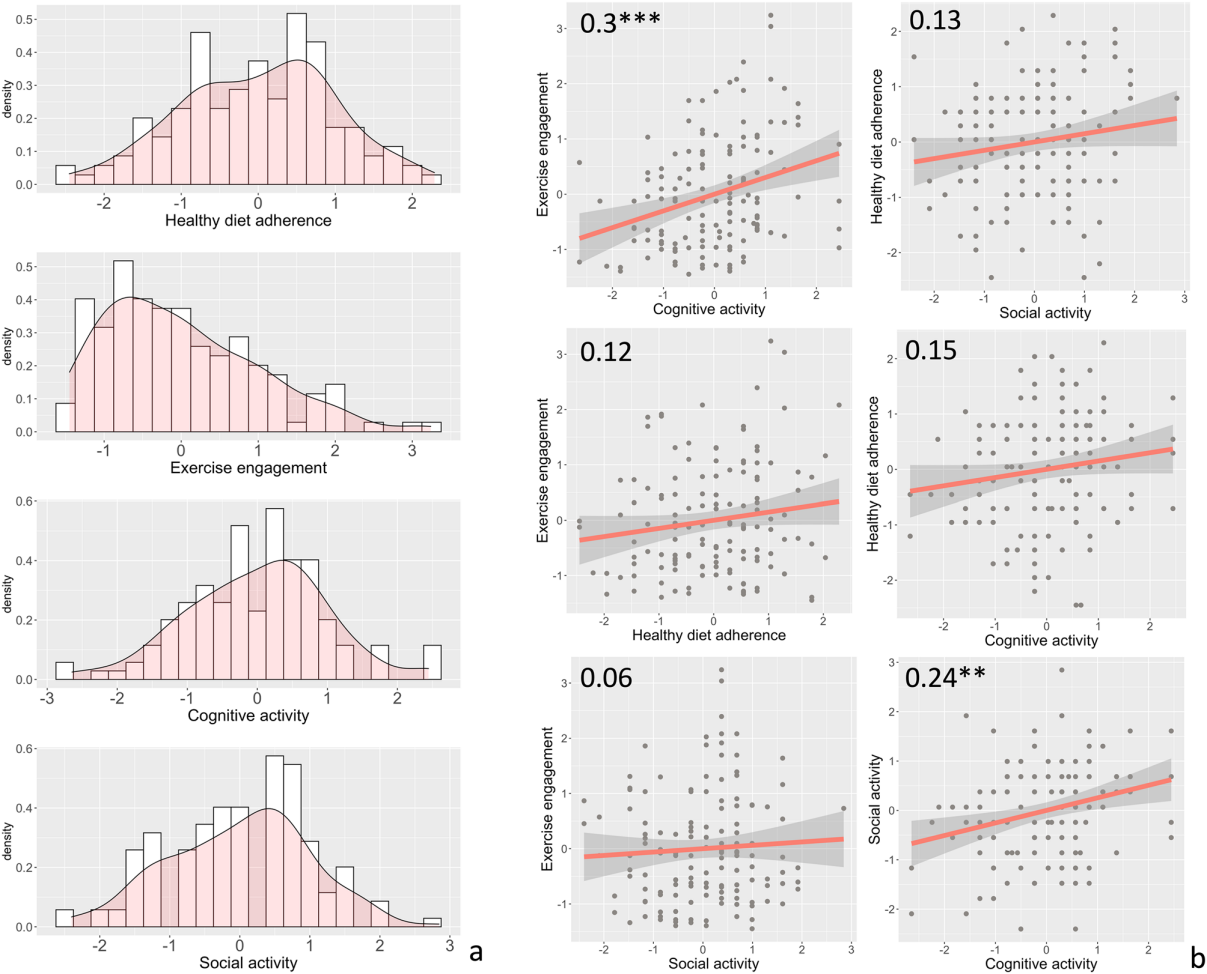
The distribution of each lifestyle variable (**a**). Scatterplots of the correlations between each pair of lifestyle variables (**b**). correlation coefficients and significance were displayed on each correlation pair (***p < 0.001, **p < 0.01, *p < 0.05).

There are 139 participants included in the k-means analysis (age = 66.53 ± 5.04; 47 males; educational years = 15.50 ± 3.46; 48 *APOE4* carriers). The distribution of each lifestyle habit and their correlation with each other are displayed in Fig. 2a. Spearman correlation was examined for each pair of lifestyle variables and the scatter plots are displayed in Fig. 2b. Cognitive activity engagement was significantly correlated with exercise engagement (*r* = 0.30, *p* = 0.001 FDR-corrected) and social activity engagement (*r* = 0.24, *p* = 0.01 FDR-corrected). While diet was not statistically associated with the other variables, a non-significant positive correlation between these lifestyle factors and all the other factors was observed.

### Lifestyle phenotyping (K-means clustering) results

**Figure 3:**
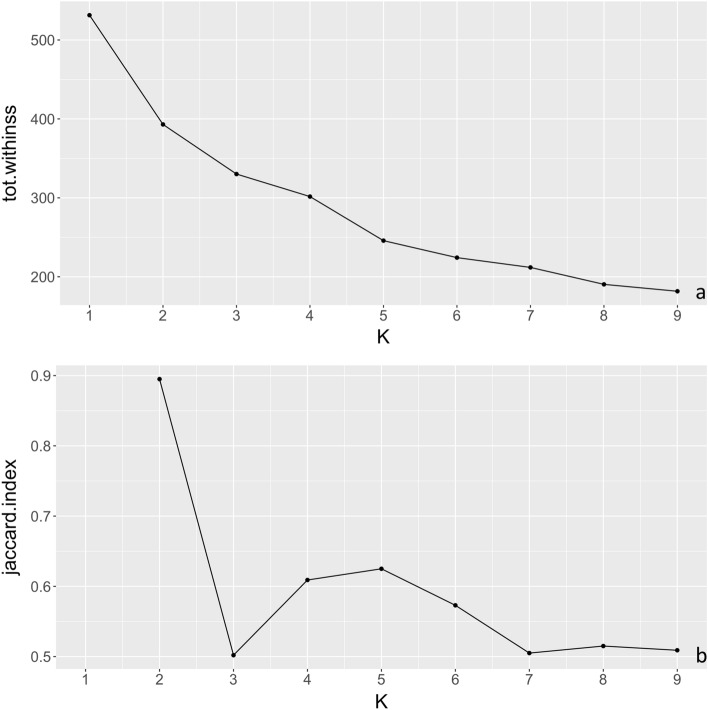
The total within sum of squares (**a**) and Jaccard index across different k (**b**), measures of cluster variability and stability respectively.

**Figure 4:**
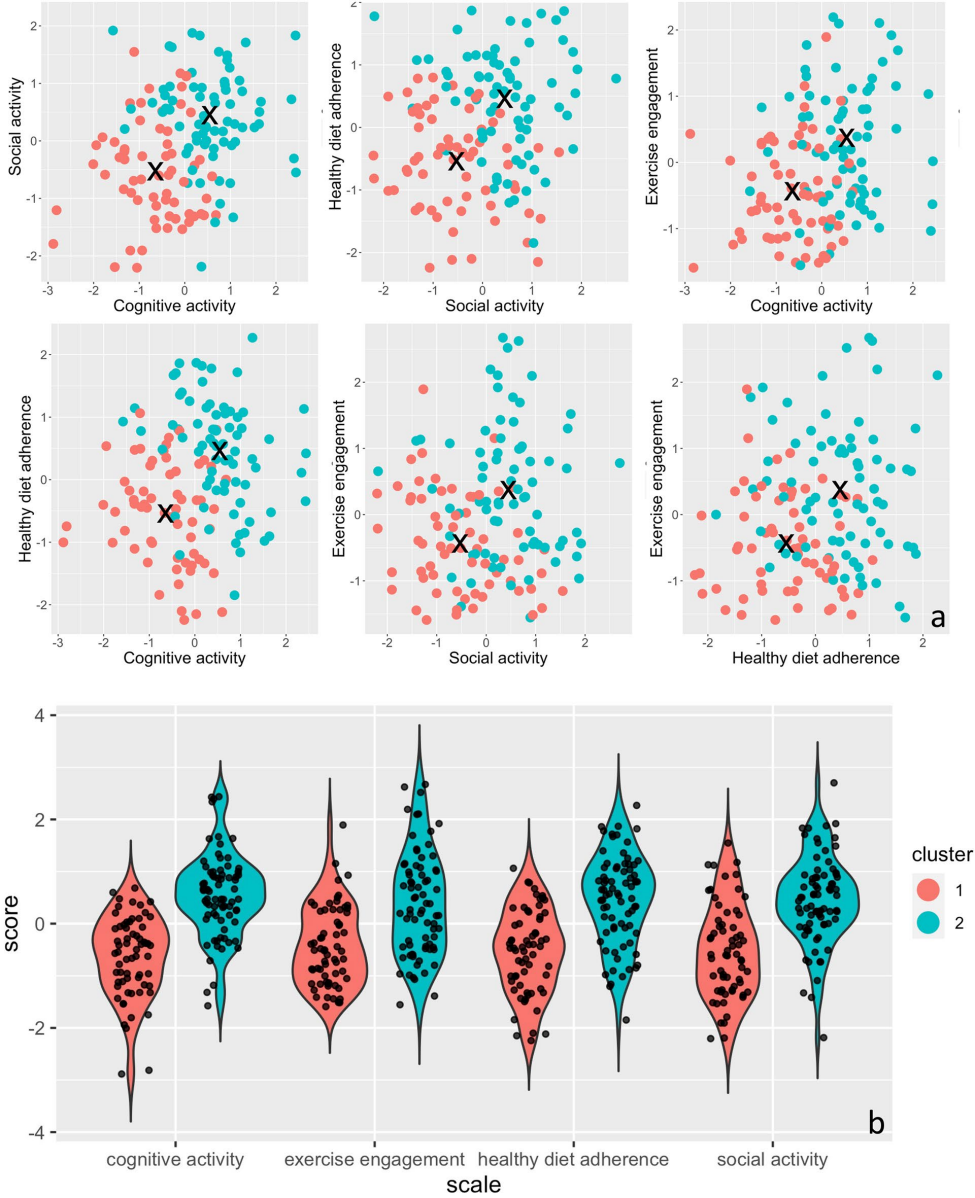
Scatterplots for cluster = 2 among pairs of lifestyle variables (**a**). The location of black cross (X) indicates the centroid of each cluster. Mean of each lifestyle variable for the two groups derived from k-means clustering (**b**). Positive scores represent greater adherence to healthy lifestyle variable. Participants in Cluster 2 showed higher levels of engagement across each health behaviour (AL group) compared to participants in Cluster 1 (NAL group).

The within cluster sum of squares showed an ‘elbow’ at *k* = 2, as it decreased with a steeper slope from *k* = 1 to 2, compared with further increments in *k* (Fig. 3a). Additionally, the highest Jaccard index appeared at *k* = 2 (i.e., 0.90 for cluster1, 0.89 for cluster2), compared to clustering solutions with a higher *k* (from 2 to 9; Fig. 3b). Therefore, the solution of two clusters provided the best fit for the current k-means analysis. The scatter plots of pairs of lifestyle variables across all k values (i.e., from 1 to 9) are displayed in Supplementary Fig. S(1). Scatterplots allow visualization of the different centers (Fig. 4a). For the final two-cluster solution, we separately examined the two groups’ lifestyle variables and found the two groups represented adherence and non-adherence to healthy lifestyle choices (Fig. 4b).

**Figure 5:**
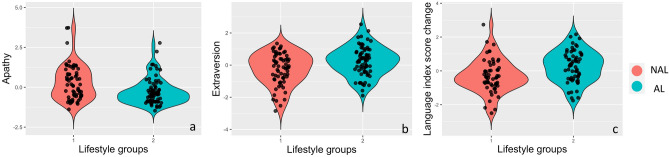
Group differences in apathy (**a**), extraversion (**b**), and language (**c**) index change. Green represents the AL group, and red represents NAL group.

The demographic information and lifestyle engagement for both groups separately are summarized in Supplementary Table S(1). We used psychosocial variables (i.e., depression, anxiety, stress, apathy, purpose of life, big five personality), and cognitive change across 4 years (i.e., RBANS change scores for five dimensions from 2016 to 2019) to validate that the two clusters reflect meaningful behavioral patterns that extend beyond lifestyle choices. The results showed that the group with non-adherence healthy lifestyle (NAL) had a higher score in apathy (*t* = 3.25, *p* = 0.007 FDR corrected; Fig. 5a) and a lower score in Big Five extraversion subscale score (*t* = − 3.0, *p* = 0.01 FDR corrected; Fig. 5b) than the group with adherence to healthy lifestyle (AL) group. NAL group also showed more longitudinal decline in language index change (*t* = − 0.31, *p* = 0.015 FDR corrected; Fig. 5c).

### Classification results

**Figure 6:**
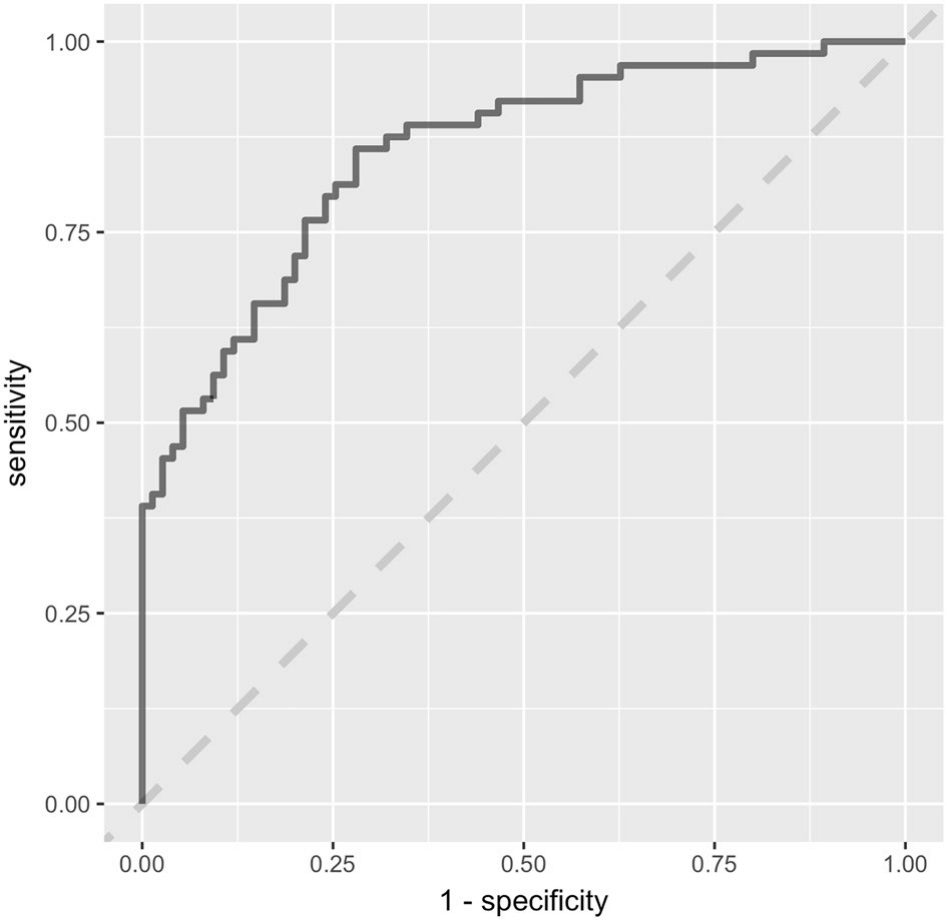
ROC for linear SVM classification model.

After feature reduction filtering, 255 brain imaging features were chosen as the final set of imaging features to be included in the classification model with 252 functional features and 3 structural features. The linear SVM model successfully classified each cluster assignment (*p* < 0.001; accuracy = 0.75, sensitivity = 0.72, specificity = 0.77, AUC = 0.87; ROC see Fig. 6). The non-linear SVM model and random forest model produced similar results (Supplementary Fig. 3).

### Feature importance

**Figure 7:**
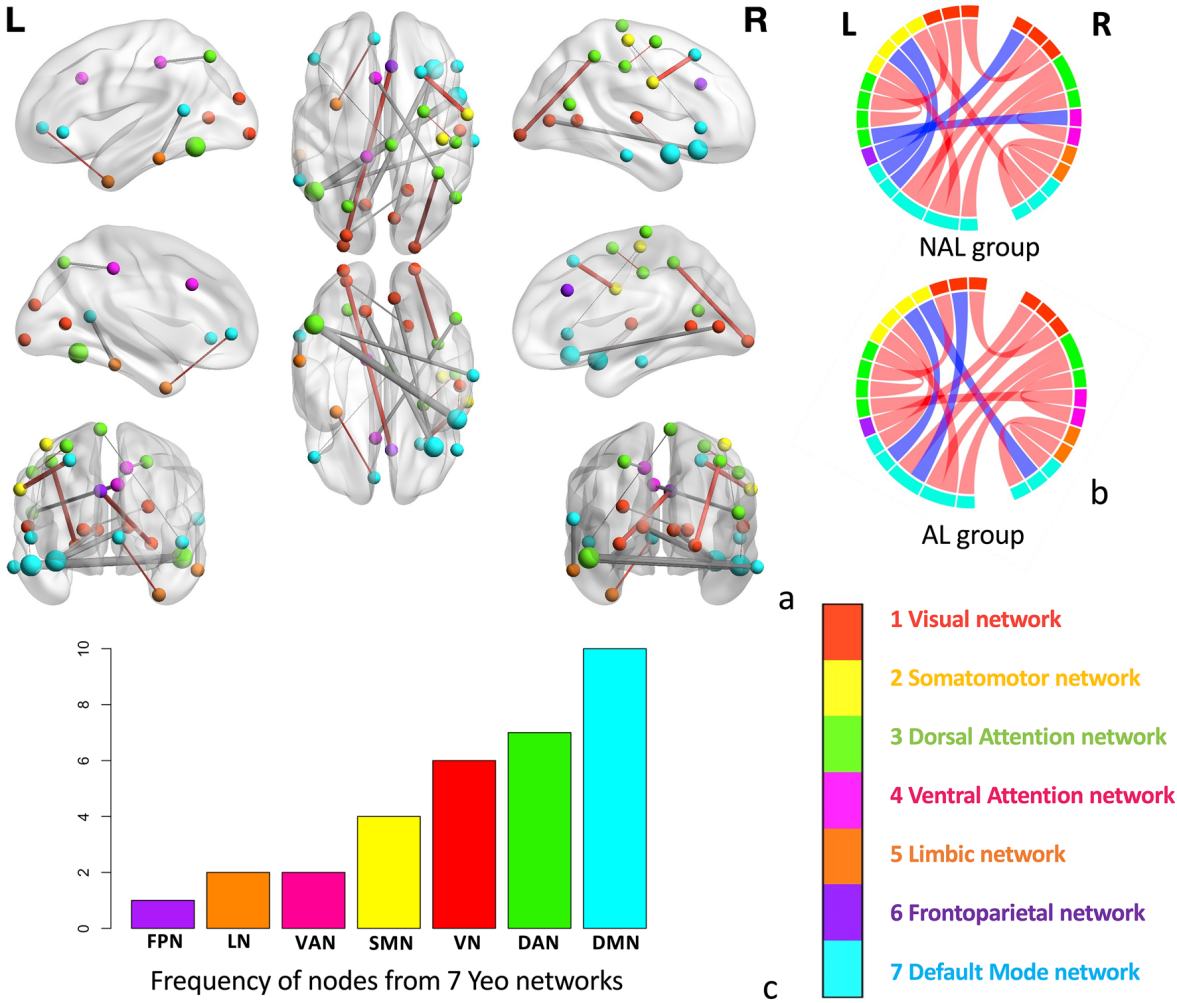
Important functional features. (**a**) Anatomical map of important features and their relative importance values. Greater node size indicates higher frequency of this node appearing in this list. Thicker edge indicates greater importance value of the connection. Red indicates greater connectivity values in AL group, and gray indicates greater connectivity values in NAL group. (**b**) Functional connectivity directionality in both groups. Red indicates positive mean connectivity across subgroups, and blue indicates negative connectivity across subgroups. (**c**) Frequency of nodes from each intrinsic resting-state network (i.e., Yeo 7 networks)^33^ in the list of important features. VN (Visual Network), SMN (Sensorimotor Network), DAN (Dorsal Attention Network), VAN (Ventral Attention Network), LN (Limbic Network), FPN (Frontoparietal Network), DMN (Default Mode Network).

The features that were the most important to the classification model are displayed in Fig. 7. The functional connections that are important for the classification model were mostly between-network and cross-hemisphere. The importance value of each feature is displayed in Table 1 and mapped on the atlas in Fig. 7a. The connectivity direction for both AL and NAL groups are displayed in Fig. 7b. Nodes that showed higher importance are distributed mainly in the default mode network, dorsal attention network, sensorimotor network and visual network (Fig. 7c).

**Table 1:** The list of connectivity features that were most important to the model.

Node1	Node2	Importance
R SMN_4	R DMN_PFCdPFCm_3	0.1091442
L VN_4	R FPN_PFCmp_1	0.10860528
R VN_4	R DAN_Post_3	0.10725516
L LN_TempPole_1	L DMN_PFC_3	0.0948649
R DAN_Post_2	R DAN_FEF_1	0.09024551
R SMN _1	R DMN_Temp_2	0.08765949
R SMN _6	R DMN_PFCv_2	− 0.0880574
L DMN_PFC_2	R SMN_8	− 0.0891813
L VN_8	R VN_6	− 0.0937739
L DAN_Post_3	L SN_Med_2	− 0.0974918
L SN_Med_1	R DAN_Post_1	− 0.103225
L DAN_Post_1	R DMN_Temp_1	− 0.1045191
R VN_5	R DMN_PFCv_1	− 0.1048217
L LN_TempPole_2	L DMN_Par_1	− 0.1053817
L VN_6	R DMN_PFCv_1	− 0.1130139
L DAN_Post_1	R DMN_Temp_2	− 0.1283484

The values of importance are displayed in the third column. Positive values indicate that the corresponding feature has greater connectivity values in AL group, and negative values indicate that the corresponding feature has greater connectivity values in NAL group. VN (Visual Network), SMN (Sensorimotor Network), DAN (Dorsal Attention Network), VAN (Ventral Attention Network), LN (Limbic Network), FPN (Frontoparietal Network), DMN (Default Mode Network). For more information about abbreviations please see Schaefer atlas documentation: https://github.com/ThomasYeoLab/CBIG/tree/master/stable_projects/brain_parcellation/Schaefer2018_LocalGlobal/Parcellations.

## Discussion

In the current study, we identified two lifestyle phenotypes among older adults at-risk for AD and examined the neurobiological distinction between these two phenotypes. We applied rigorous data-driven machine learning methods, an important step towards generalizable findings and precision neurology. We have two major findings in this study. Firstly, the data-driven clustering approach revealed two distinct phenotypes that adhere to a healthier lifestyle and a less healthy lifestyle in all four categories of lifestyle habits. We found that older adults engaged in protective lifestyle behaviors across multiple domains, rather than in isolation. Between-group differences were also verified in increased extraversion, lower apathy levels, and more preserved cognitive change over time in adherence group. Secondly, the classification model identified functional connectivity features that successfully differentiated the two lifestyle phenotypes. Most features represented between-network functional connectivity, which revealed a distributed set of neural features related to lifestyle choices. The non-adherent lifestyle group (i.e., NAL group) showed a larger number of features representing greater between-network and cross-hemisphere functional connectivity than adherent lifestyle group (i.e., AL group).

We validated patterns in our data that showed two distinct sub-groups of people who either adhere well to all four lifestyle habits or adhered poorly to all four habits. The results indicated potential covarying factors across different lifestyle behaviors, which was under-examined in prior literature. Importantly, we found convergent validity for these two clusters using psychosocial and cognitive variables independent from the input of the clustering analysis. These findings converge with prior research. For example, older adults with higher extraversion and agreeableness were found to have both greater social networks and more moderate physical activity engagement^34,35^, which is consistent with our finding of the AL group having higher extraversion scores. There might be also a bidirectional relationship between executive function and health behaviors, such as physical activity^36^. Consistently, our results showed difference in language index changes between AL and NAL groups, and the semantic fluency subtest was driving this difference (*t* = − 3.14, *p* = 0.002; see Supplementary Fig. S(2)), which loads on executive control components^37^. Therefore, the distinction we observed across two multidomain lifestyle profiles indicates a potential set of psychological and neurobiological factors shared by many health behaviors, and at the same time provides validation that the two phenotypes found in our data-driven approach reflect ecologically valid patterns in the data.

We identified the neural features that differentiated individuals with differences in multidomain lifestyles using a classification prediction model. Features with higher weight and consistency for the prediction model were all between-network functional connectivity features, some of which showed greater functional connectivity in NAL group, while others showed greater functional connectivity in AL group. Nodes in temporal regions and prefrontal regions belonging to the DMN and DAN were found most frequently in these discriminative features (Fig. 7c). Previous studies also found that functional connectivity in DMN and attentional networks predicted adherence to an exercise intervention and a mental training programs^38,39^. Meanwhile, both DMN and DAN play an important role in supporting executive function^40,41^, which were found to contribute to voluntary physical activity engagement^42^. One potential psychological mechanism behind this relationship is that executive function facilitates initiating and adhering to health behaviors through “temporal self-regulation”^36^, with three important determinants for physical activity engagement: physical activity prepotency, intention, and executive function^43^. Combining the neurobiological distinction in nodes involving DMN and DAN and behavioral difference in verbal fluency between AL and NAL in our findings, it suggests that this connection between executive control and physical activity may generalize to multidomain lifestyles, and it might be a promising attempt to examine this theoretical framework across multiple lifestyle behaviors in the future.

Most of the features with stably high importance represented enhanced between network connectivity. More between-network features showed increased functional connectivity values in the NAL group compared to the AL group (9 vs 5). Specifically, individuals from the NAL group showed greater positive correlation or less anti-correlation between DMN and other task-positive networks (e.g., DAN, SMN) than the AL group. Studies have found that higher cross-network connectivity and lower within-network connectivity are associated with cognitive decline in healthy older adults^44,45^. A less segregated functional brain was also associated with AD symptoms^46,47^. Previous studies also identified the decreased anti-correlated relationship between DMN and task-positive networks (e.g. DAN, FPN) in both normal aging population and AD patients^48–51^. Regarding lifestyle, older adults who engaged more in physical and cognitive activity, showed greater modularity, a measure of network distinctiveness^12^. Therefore, in a summary, greater functional connectivity between task-negative and task-positive networks in the NAL group might suggest a more vulnerable connectivity status linked with lifestyle behaviors.

The final classification model included a majority of functional connectivity and only a few structural features after feature selection, and the most important and consistent features all consisted of functional connectivity variables. This indicates that the relationship between multidomain lifestyle habits and brain health might be primarily supported by cognitive reserve or functional plasticity (i.e., a more adaptable functional brain with higher efficiency and flexibility for cognitive processing) rather than brain reserve (i.e., the structural characteristics of a brain that copes with pathology and function loss)^52^. Although this observation is not necessarily consistent with some previous multidomain lifestyle studies that identified associations between lifestyle and selective aspects of brain structure^19,20,53^, the discrepancy may result from different methods and populations. The current study applied a data driven method by including all neural features in the same model, while other studies examined structural and functional features separately. Moreover, all of our participants were at high risk for AD. The structural characteristic in the current sample may already differ from other studies. Some high importance nodes in DMN in middle temporal gyrus (i.e., R DMN_Temp_1 and R DMN_Temp_2) and inferior frontal (i.e., R DMN_PFCv_2)^32^ overlapped with the rich club hubs that showed different nodal efficiency between AD patients and healthy older adults from a previous report^54^, indicating potential pre-symptomatic AD-related alternation in our current sample. Therefore, it is important to focus more attention on high AD-risk samples, and regions and networks that are vulnerable to AD pathology.

There are some limitations in the current study. First, we had a relatively small sample size, which may cause increased bias for the results accuracy^55^ and low stability^56^ for machine learning models. Accordingly, we interpret with caution individual features from the machine learning models. However, our study did show biological distinction among individuals with different lifestyle profiles, which provides insight into lifestyle-brain associations. Second, this was a cross-sectional design. We identified features linked to lifestyle behaviors, but we cannot establish any causal relationship between functional connectivity and lifestyles in the current study. Analyses using longitudinal or interventional study designs are needed in future research to further disentangle the bidirectional relationship between brain health and lifestyle habits. Third, the collection time of different variables did not always occur at the same time. The neuroimaging data were collected one or two years earlier than the lifestyle variables, and exercise variables were collected one year earlier than the other lifestyle variables. We hypothesize that lifestyle habits stay consistent across these years, particularly among older adults, but we cannot exclude the potential confounding of behavioral change. Lastly, the sample consists of a majority of females, which decreased the generalizability for the results. Nevertheless, we controlled the for sex by including it as a covariate in all models in the study.

In conclusion, we identified two phenotypes of lifestyle participation and the neurobiological distinction between them. The prediction model suggested an association between multidomain lifestyles and functional connectivity features in older adults at high risk for AD. Studies with larger samples and more diverse populations are needed to further examine brain-lifestyle relationships. Future studies should allocate more attention to investigating the psychosocial and cognitive factors that promote protective lifestyles, from real-world adherence to response and behavioral change following interventions.

## Supplementary Information

Supplementary Information.

## Data Availability

This dataset belongs to the Pre-symptomatic Evaluation of Novel or Experimental Treatments for Alzheimer’s disease (PREVENT-AD) program data internal release 6.0. Part of the data from PREVENT-AD program is accessible to all public through openpreventad.loris.ca and more complete data are available to researchers/physician through registeredpreventad.loris.ca. Information about data access is available at: https://prevent-alzheimer.net/?page_id=1760&lang=en.

## References

[1] Gustavsson, A. *et al.* Global estimates on the number of persons across the Alzheimer’s disease continuum. *Alzheimer’s & Dementia* n/a, (2022). 10.1002/alz.12694 35652476

[2] Alzheimer’s Association. 2022 Alzheimer’s disease facts and figures. https://www.alz.org/media/Documents/alzheimers-facts-and-figures.pdf (2022).

[3] Livingston G (2020). Dementia prevention, intervention, and care: 2020 report of the Lancet Commission. Lancet.

[4] Shatenstein B (2012). Diet quality and cognition among older adults from the NuAge study. Exp. Gerontol..

[5] Kramer AF, Colcombe S (2018). Fitness effects on the cognitive function of older adults: A meta-analytic study—Revisited. Perspect Psychol. Sci..

[6] Wilson RS (2013). Life-span cognitive activity, neuropathologic burden, and cognitive aging. Neurology.

[7] Seeman TE, Lusignolo TM, Albert M, Berkman L (2001). Social relationships, social support, and patterns of cognitive aging in healthy, high-functioning older adults: MacArthur studies of successful aging. Health Psychol..

[8] Cass SP (2017). Alzheimer’s disease and exercise: A literature review. Curr. Sports Med. Rep..

[9] Singh B (2014). Association of mediterranean diet with mild cognitive impairment and Alzheimer’s disease: A systematic review and meta-analysis. J. Alzheimer’s Dis..

[10] Wilson RS, Scherr PA, Schneider JA, Tang Y, Bennett DA (2007). Relation of cognitive activity to risk of developing Alzheimer disease. Neurology.

[11] de Frias CM, Dixon RA (2014). Lifestyle engagement affects cognitive status differences and trajectories on executive functions in older adults. Arch. Clin. Neuropsychol..

[12] Soldan A (2021). Association of lifestyle activities with functional brain connectivity and relationship to cognitive decline among older adults. Cereb. Cortex.

[13] Wirth M, Haase CM, Villeneuve S, Vogel J, Jagust WJ (2014). Neuroprotective pathways: Lifestyle activity, brain pathology, and cognition in cognitively normal older adults. Neurobiol. Aging.

[14] Andrieu S, Coley N, Lovestone S, Aisen PS, Vellas B (2015). Prevention of sporadic Alzheimer’s disease: Lessons learned from clinical trials and future directions. Lancet Neurol..

[15] Kivipelto M, Mangialasche F, Ngandu T (2018). Lifestyle interventions to prevent cognitive impairment, dementia and Alzheimer disease. Nat. Rev. Neurol..

[16] Talwar P (2016). Dissecting complex and multifactorial nature of Alzheimer’s disease pathogenesis: A clinical, genomic, and systems biology perspective. Mol. Neurobiol..

[17] Tamers SL (2011). The association between worksite social support, diet, physical activity and body mass index. Prev. Med..

[18] Simoes EJ (1995). The association between leisure-time physical activity and dietary fat in American adults. Am. J. Public Health.

[19] Bittner N (2021). When your brain looks older than expected: Combined lifestyle risk and BrainAGE. Brain Struct. Funct..

[20] Schreiber S (2016). Impact of lifestyle dimensions on brain pathology and cognition. Neurobiol. Aging.

[21] National Academies of Sciences, E. *Preventing Cognitive Decline and Dementia: A Way Forward*. (2017). 10.17226/24782. 28650595

[22] Tremblay-Mercier J (2021). Open science datasets from PREVENT-AD, a longitudinal cohort of pre-symptomatic Alzheimer’s disease. NeuroImage Clin..

[23] Sheikh JI, Yesavage JA (1986). Geriatric depression scale (GDS): Recent evidence and development of a shorter version. Clin. Gerontol. J. Aging Mental Health.

[24] Pachana NA (2007). Development and validation of the Geriatric anxiety inventory. Int. Psychogeriatr..

[25] Lovibond PF, Lovibond SH (1995). The structure of negative emotional states: Comparison of the depression anxiety stress scales (DASS) with the beck depression and anxiety inventories. Behav. Res. Ther..

[26] Marin RS, Biedrzycki RC, Firinciogullari S (1991). Reliability and validity of the apathy evaluation scale. Psychiatry Res..

[27] John, O. P., Donahue, E. M. & Kentle, R. L. Big Five Inventory. (1991) 10.1037/t07550-000

[28] Randolph C, Tierney MC, Mohr E, Chase TN (1998). The repeatable battery for the assessment of neuropsychological status (RBANS): Preliminary clinical validity. J. Clin. Exp. Neuropsychol..

[29] Esteban O (2019). fMRIPrep: A robust preprocessing pipeline for functional MRI. Nat. Methods.

[30] Whitfield-Gabrieli S, Nieto-Castanon A (2012). Conn: A functional connectivity toolbox for correlated and anticorrelated brain networks. Brain Connect..

[31] Van Dijk KRA (2010). Intrinsic functional connectivity as a tool for human connectomics: Theory, properties, and optimization. J. Neurophysiol..

[32] Schaefer A (2018). Local-global parcellation of the human cerebral cortex from intrinsic functional connectivity MRI. Cereb. Cortex.

[33] Thomas Yeo BT (2011). The organization of the human cerebral cortex estimated by intrinsic functional connectivity. J. Neurophysiol..

[34] Artese A, Ehley D, Sutin AR, Terracciano A (2017). Personality and actigraphy-measured physical activity in older adults. Psychol. Aging.

[35] Rollings J, Micheletta J, Van Laar D, Waller BM (2022). Personality traits predict social network size in older adults. Pers. Soc. Psychol. Bull..

[36] Allan JL, McMinn D, Daly M (2016). A bidirectional relationship between executive function and health behavior: Evidence, implications, and future directions. Front. Neurosci..

[37] Shao Z, Janse E, Visser K, Meyer AS (2014). What do verbal fluency tasks measure? Predictors of verbal fluency performance in older adults. Front. Psychol..

[38] Morris TP (2022). What can the brain tell us about older adult’s engagement in physical exercise and sedentary behaviors? 1078. Med. Sci. Sports Exerc..

[39] Saghayi M (2020). Brain network topology predicts participant adherence to mental training programs. Netw. Neurosci..

[40] Corbetta M, Shulman GL (2002). Control of goal-directed and stimulus-driven attention in the brain. Nat. Rev. Neurosci..

[41] Sambataro F (2010). Age-related alterations in default mode network: Impact on working memory performance. Neurobiol. Aging.

[42] Buckley J, Cohen JD, Kramer AF, McAuley E, Mullen SP (2014). Cognitive control in the self-regulation of physical activity and sedentary behavior. Front. Hum. Neurosci..

[43] Hall PA, Fong GT (2015). Temporal self-regulation theory: A neurobiologically informed model for physical activity behavior. Front. Hum. Neurosci..

[44] Grady C, Sarraf S, Saverino C, Campbell K (2016). Age differences in the functional interactions among the default, frontoparietal control, and dorsal attention networks. Neurobiol. Aging.

[45] Jiang R (2022). A neuroimaging signature of cognitive aging from whole-brain functional connectivity. Adv. Sci..

[46] Brier MR (2014). Functional connectivity and graph theory in preclinical Alzheimer’s disease. Neurobiol. Aging.

[47] Ewers M (2021). Segregation of functional networks is associated with cognitive resilience in Alzheimer’s disease. Brain.

[48] Esposito R (2018). Modifications in resting state functional anticorrelation between default mode network and dorsal attention network: Comparison among young adults, healthy elders and mild cognitive impairment patients. Brain Imaging Behav..

[49] Keller JB (2015). Resting-state anticorrelations between medial and lateral prefrontal cortex: Association with working memory, aging, and individual differences. Cortex.

[50] Ng KK, Lo JC, Lim JKW, Chee MWL, Zhou J (2016). Reduced functional segregation between the default mode network and the executive control network in healthy older adults: A longitudinal study. Neuroimage.

[51] Wang J (2019). Dysfunctional interactions between the default mode network and the dorsal attention network in subtypes of amnestic mild cognitive impairment. Aging.

[52] Stern Y (2020). Whitepaper: Defining and investigating cognitive reserve, brain reserve, and brain maintenance. Alzheimer’s Dementia.

[53] Bittner N (2019). Combining lifestyle risks to disentangle brain structure and functional connectivity differences in older adults. Nat. Commun..

[54] Cao R (2020). Abnormal anatomical rich-club organization and structural-functional coupling in mild cognitive impairment and Alzheimer’s disease. Front. Neurol..

[55] Vabalas A, Gowen E, Poliakoff E, Casson AJ (2019). Machine learning algorithm validation with a limited sample size. PLoS ONE.

[56] Cui Z, Gong G (2018). The effect of machine learning regression algorithms and sample size on individualized behavioral prediction with functional connectivity features. Neuroimage.

