# Supplementary material for: Resting-state MRI functional connectivity as a neural correlate of multidomain lifestyle adherence in older adults at risk for Alzheimer’s disease
Source: Sci Rep. 2023 May 9;13:7487. doi: 10.1038/s41598-023-32714-1 (PMC10170147; doi:10.1038/s41598-023-32714-1)
Supplement: Supplementary file 1 — Supplementary Information. [file 41598_2023_32714_MOESM1_ESM.docx]

**Supplementary materials**


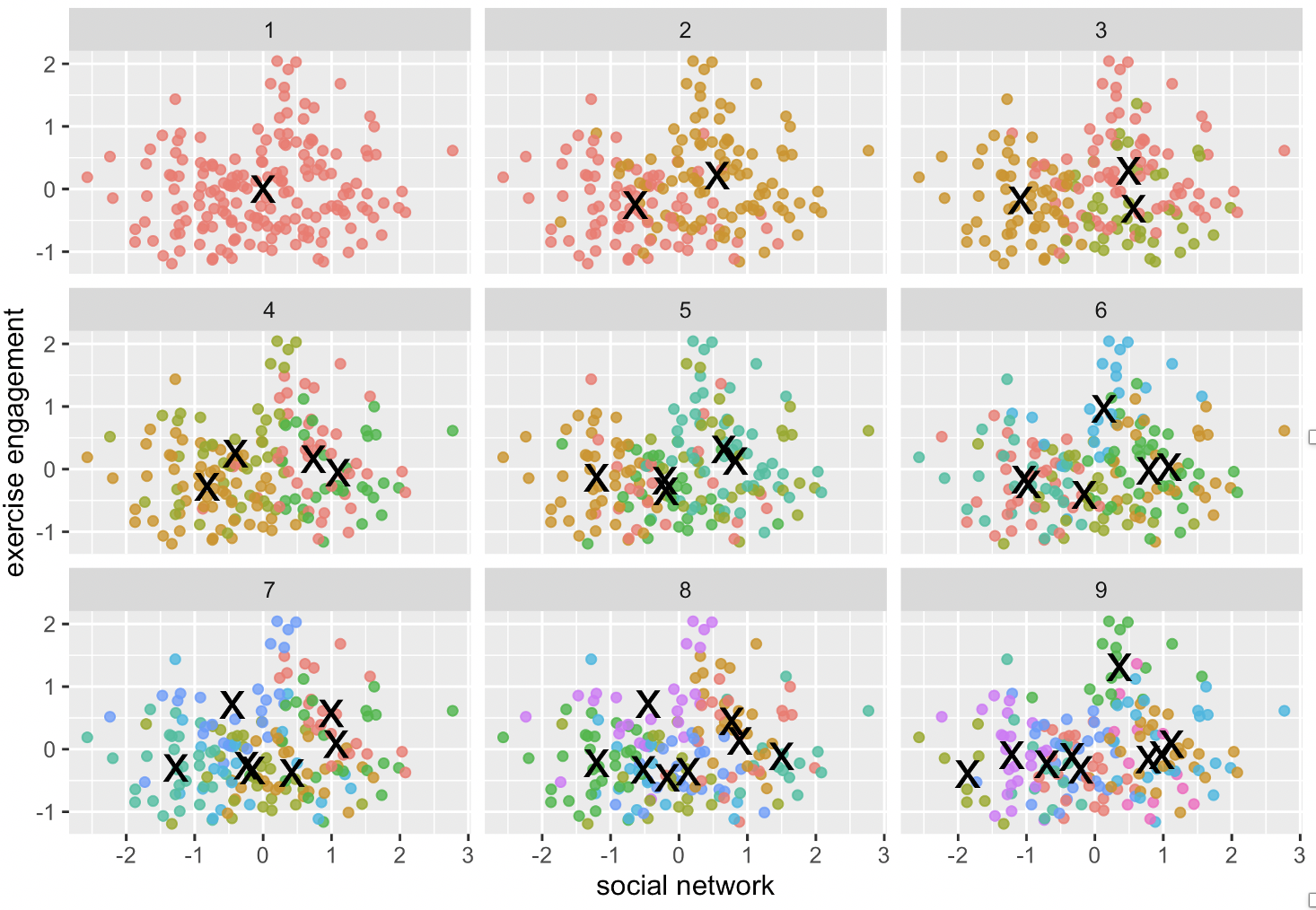

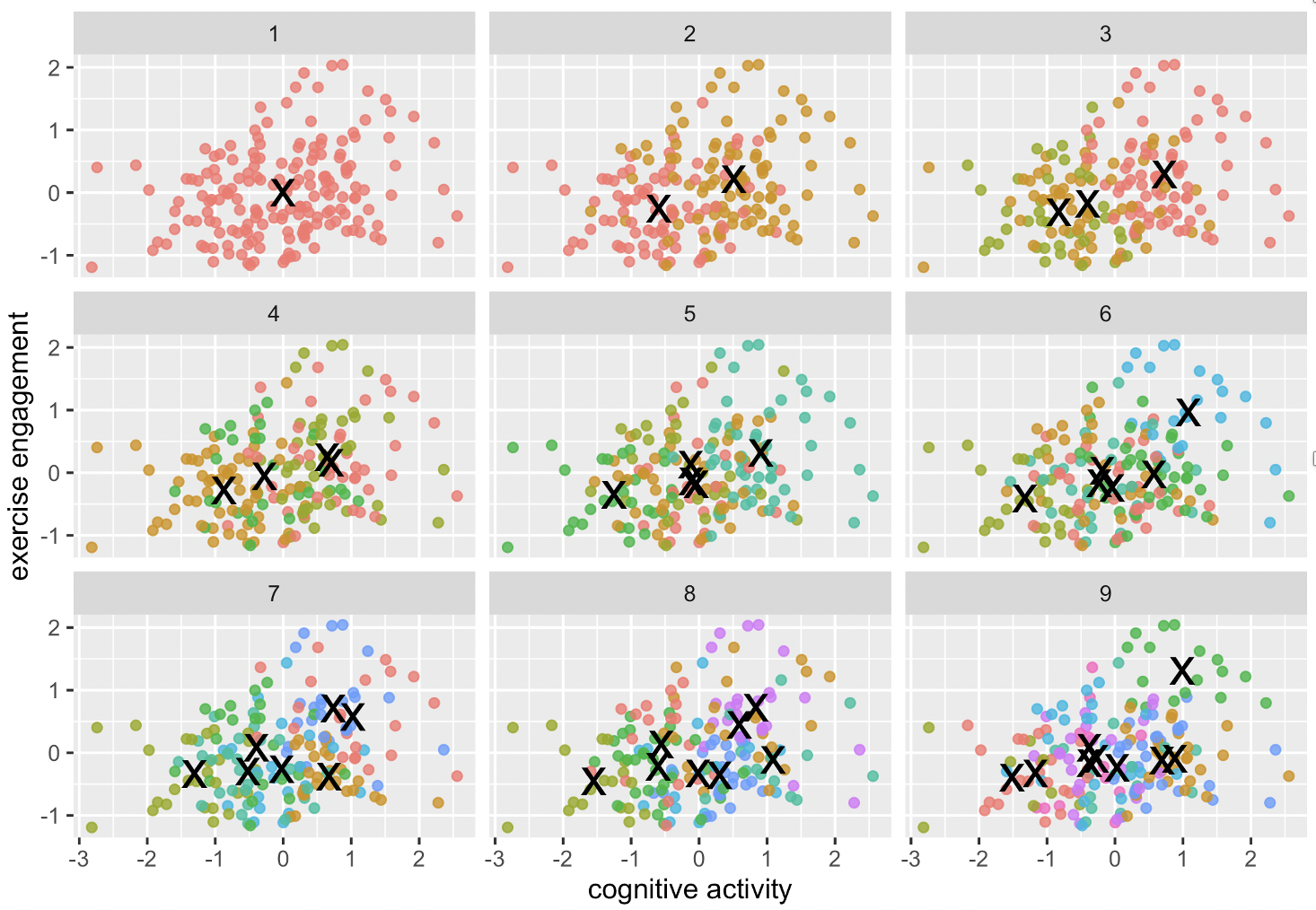

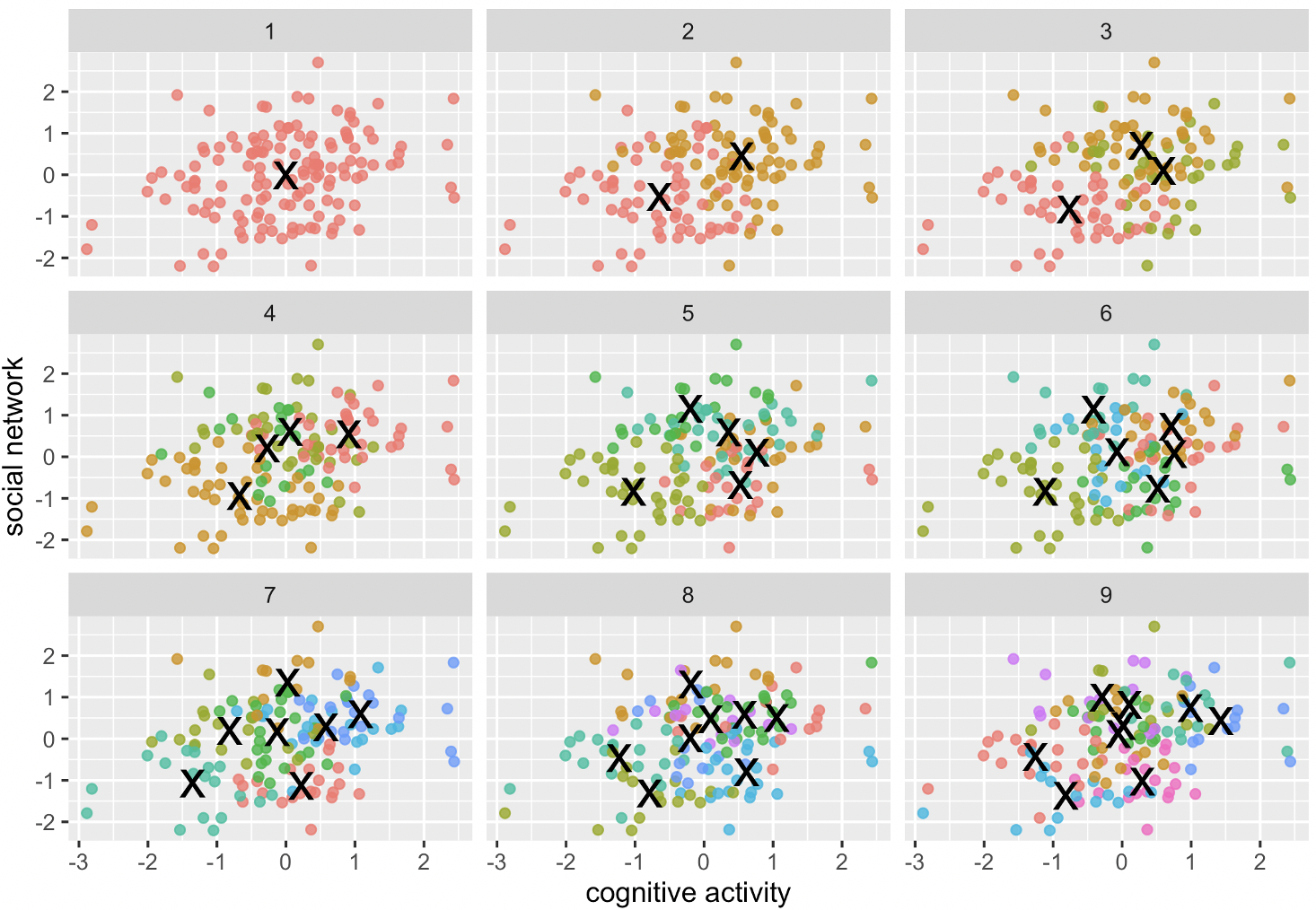

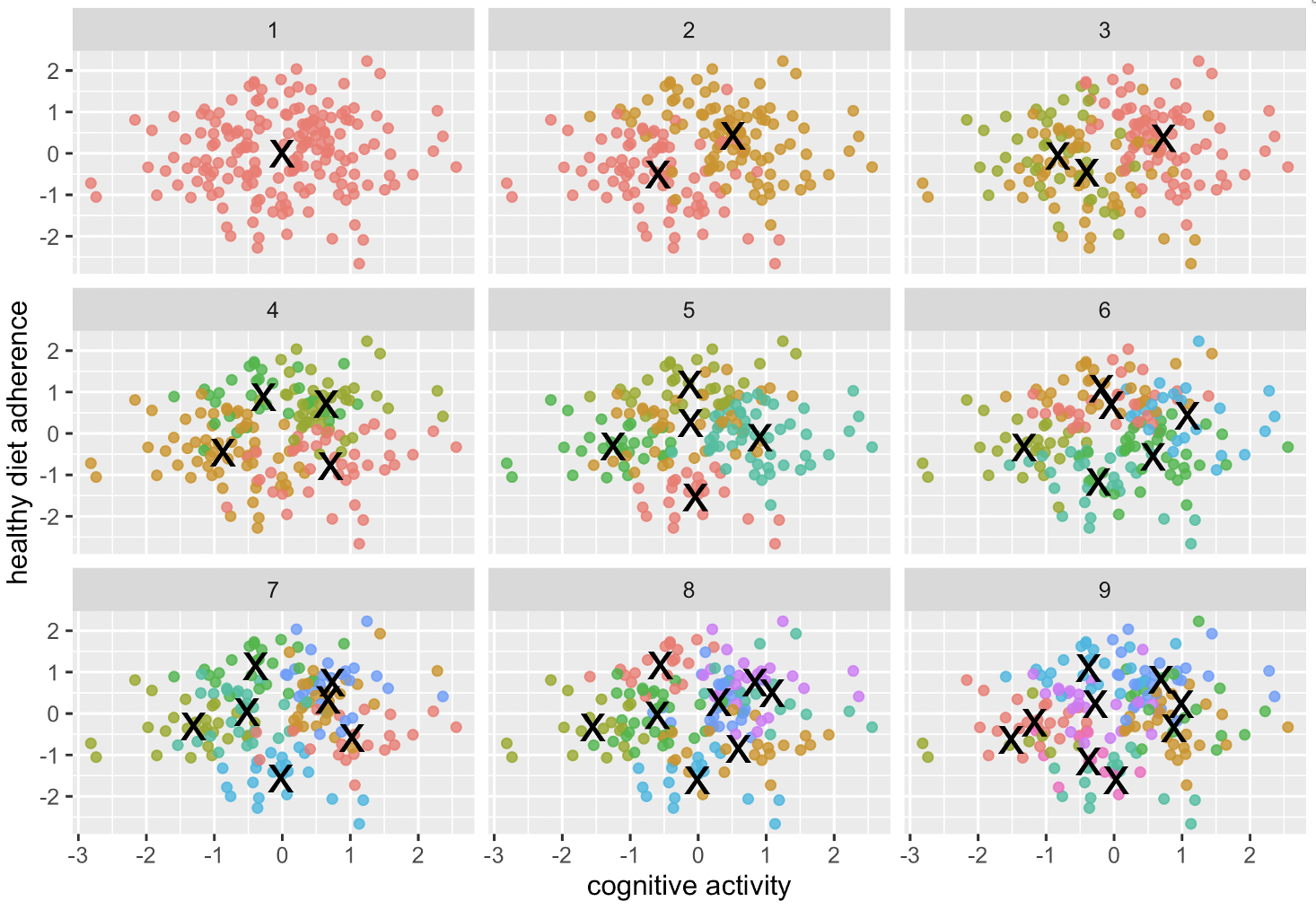

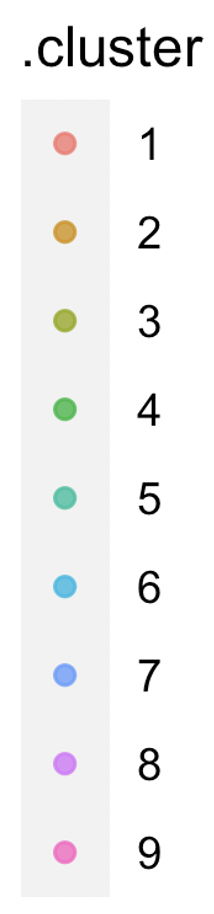

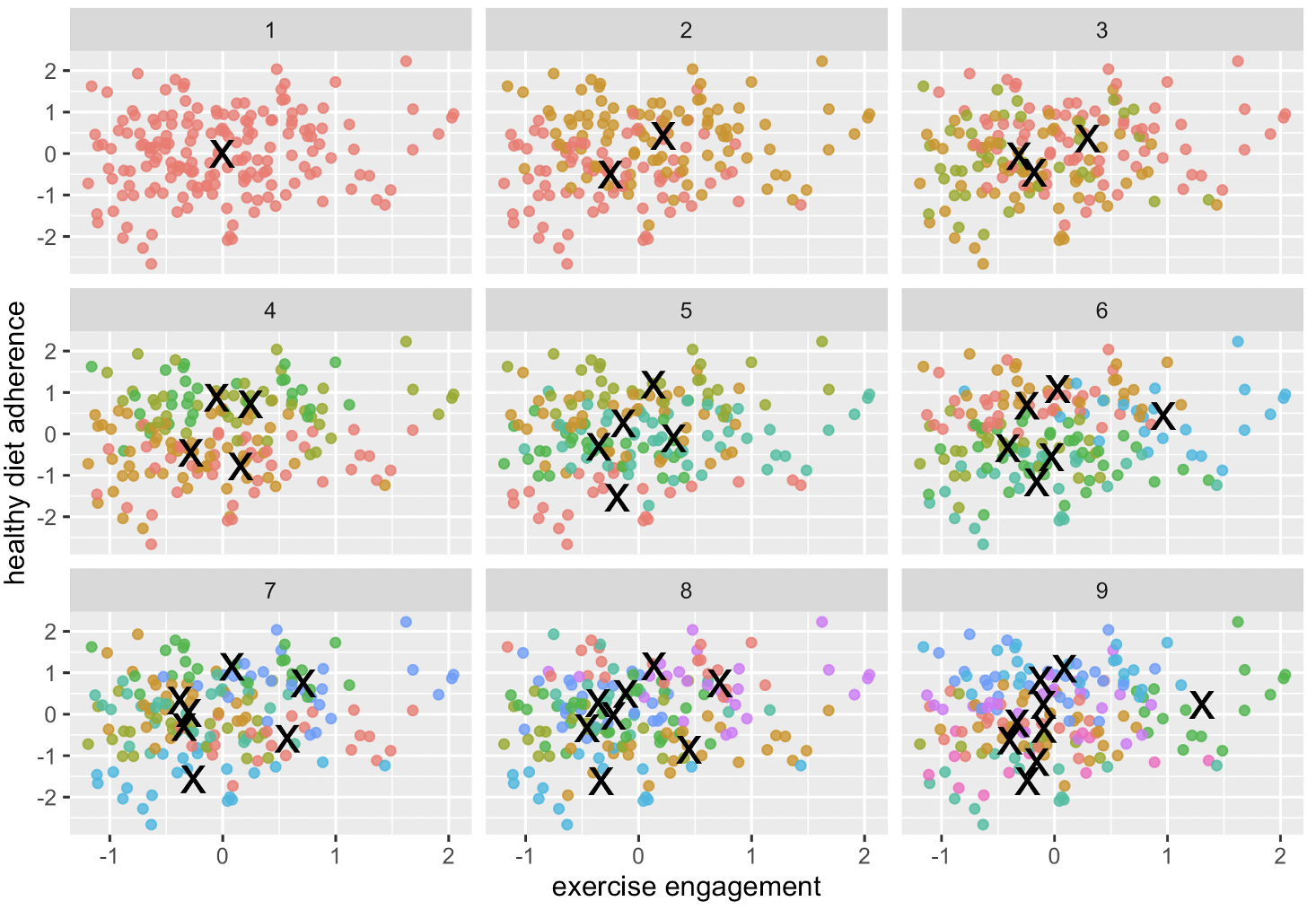

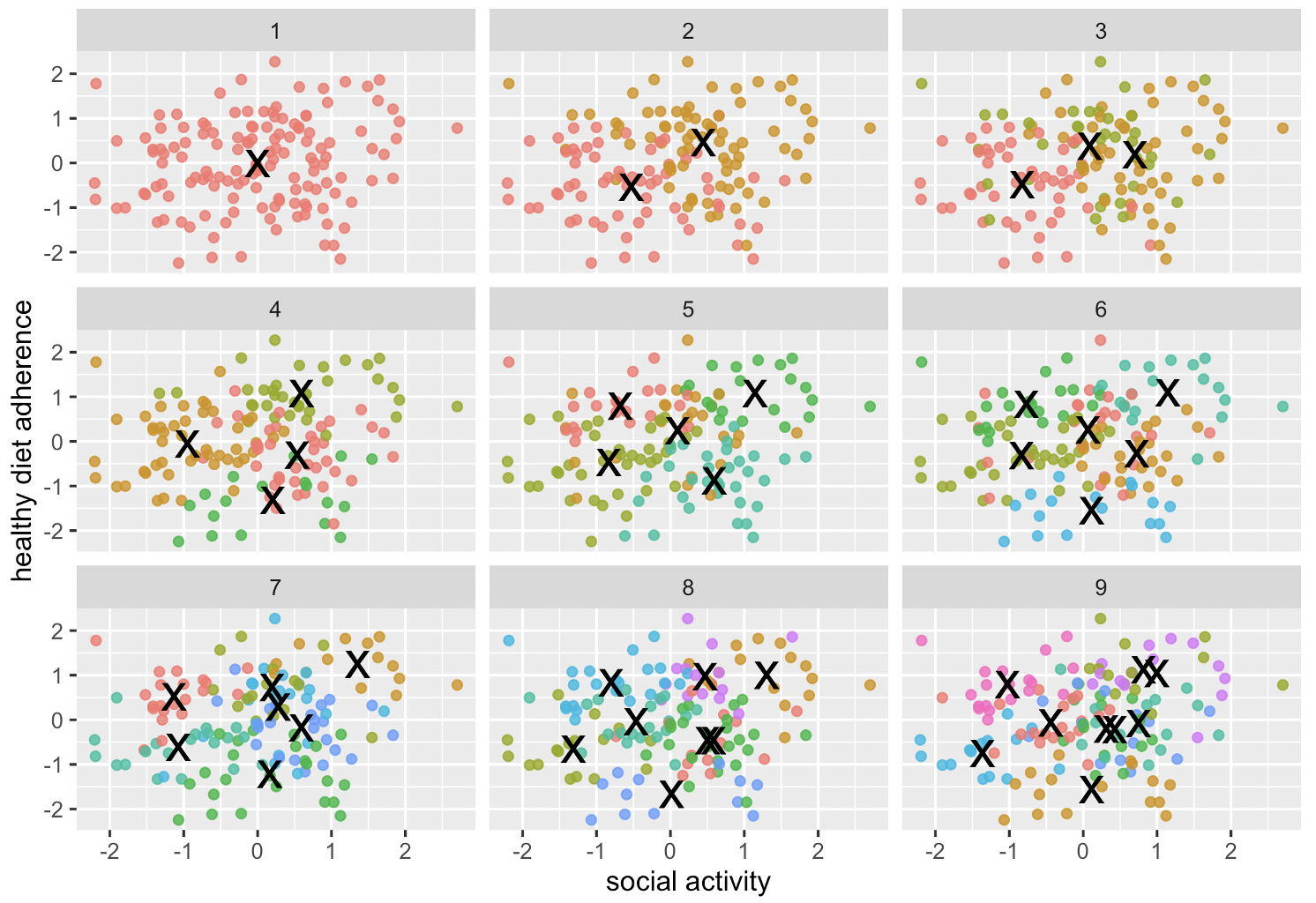


*Supplementary Figure S(1)* Scatter plots between pairs of lifestyle variables across k=1 to k=9. The color indicates individual clusters.


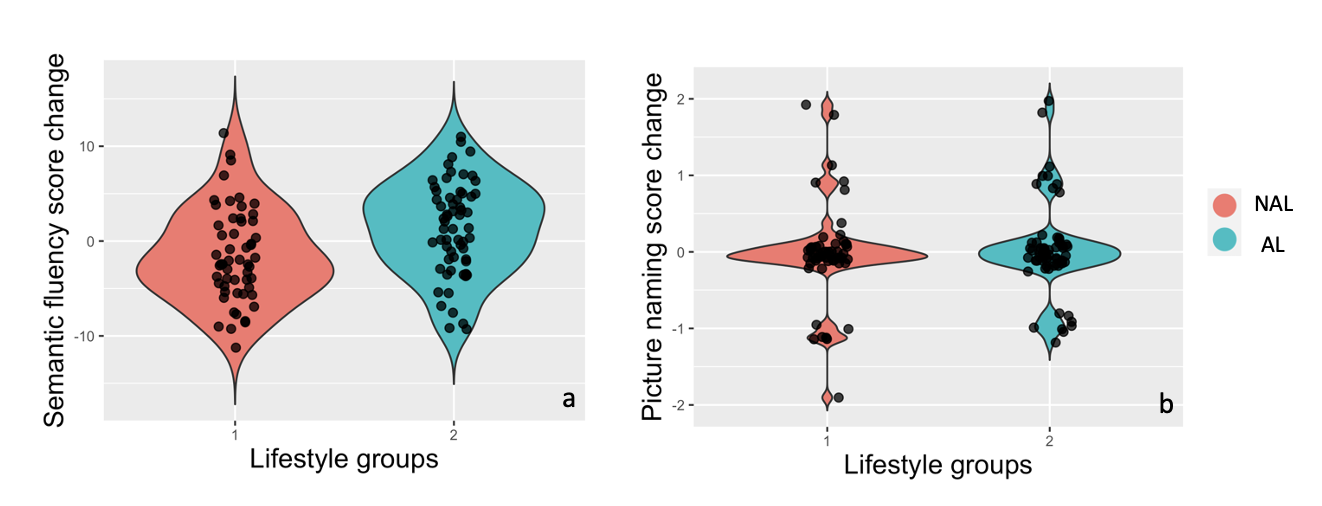


b

a

*Supplementary Figure S(2)* Group difference in semantic fluency subscale score change (a; *t*=-3.14, *p*=0.002) and picture naming subscale score change (b; *t*=-0.45, *p*=0.65).


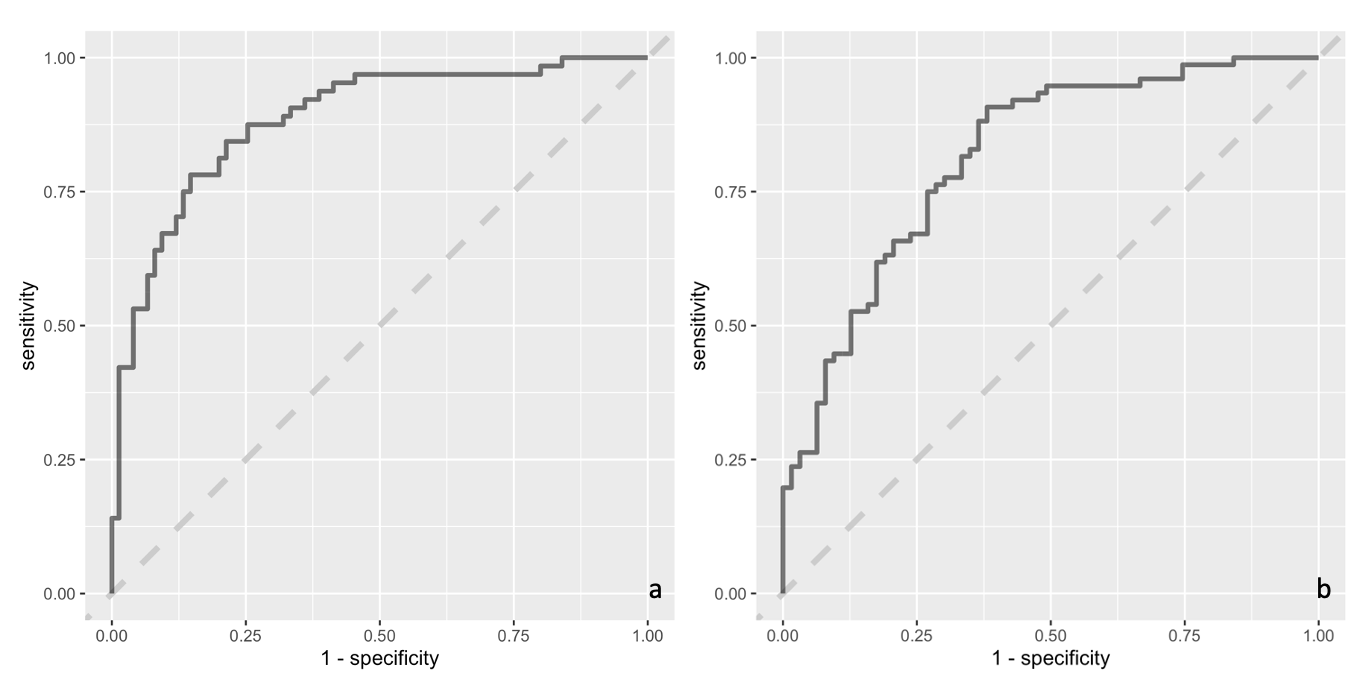


*Supplementary figure S(3).* (a) ROC for non-linear SVM classification model (*p*<0.001; accuracy=0.80, sensitivity=0.74, specificity=0.87, AUC=0.89). (b) ROC for the random forest model (*p*<0.001; accuracy=0.74, sensitivity=0.59, specificity=0.87, AUC=0.83).

|  | AL group (n=75) | NAL group (n=64) |
| --- | --- | --- |
| Age | 66.20±4.59 | 66.28±4.40 |
| Sex | 56 female | 49 female |
| Years of Education | 15.60±2.87 | 15.81±2.91 |
| APOE4 carriers | 28 carriers | 20 carriers |
| Exercise engagement score | 394.59±228.94 | 222.24±160.67 |
| Cognitive activity score | 3.58±0.62 | 2.70±0.59 |
| Healthy diet adherence score | 59.65±3.67 | 55.69±3.28 |

*Supplementary Table S(1)* Demographic information and lifestyle scores for both groups (AL and NAL).
